# Is Retinal Metabolic Dysfunction at the Center of the Pathogenesis of Age-related Macular Degeneration?

**DOI:** 10.3390/ijms20030762

**Published:** 2019-02-11

**Authors:** Thierry Léveillard, Nancy J. Philp, Florian Sennlaub

**Affiliations:** 1 Department of Genetics, Sorbonne Université, INSERM, CNRS, Institut de la Vision, 17 rue Moreau, F-75012 Paris, France; 2 Department of Pathology, Anatomy and Cell Biology, Thomas Jefferson University, Philadelphia, PA 19107, USA; Nancy.Philp@jefferson.edu; 3 Department of Therapeutics, Sorbonne Université, INSERM, CNRS, Institut de la Vision, 17 rue Moreau, F-75012 Paris, France; florian.sennlaub@inserm.fr

**Keywords:** cone photoreceptor, inflammatory macrophage, aerobic glycolysis, lactate transporter, retinal degeneration, rod-derived cone viability factor

## Abstract

The retinal pigment epithelium (RPE) forms the outer blood–retina barrier and facilitates the transepithelial transport of glucose into the outer retina via GLUT1. Glucose is metabolized in photoreceptors via the tricarboxylic acid cycle (TCA) and oxidative phosphorylation (OXPHOS) but also by aerobic glycolysis to generate glycerol for the synthesis of phospholipids for the renewal of their outer segments. Aerobic glycolysis in the photoreceptors also leads to a high rate of production of lactate which is transported out of the subretinal space to the choroidal circulation by the RPE. Lactate taken up by the RPE is converted to pyruvate and metabolized via OXPHOS. Excess lactate in the RPE is transported across the basolateral membrane to the choroid. The uptake of glucose by cone photoreceptor cells is enhanced by rod-derived cone viability factor (RdCVF) secreted by rods and by insulin signaling. Together, the three cells act as symbiotes: the RPE supplies the glucose from the choroidal circulation to the photoreceptors, the rods help the cones, and both produce lactate to feed the RPE. In age-related macular degeneration this delicate ménage à trois is disturbed by the chronic infiltration of inflammatory macrophages. These immune cells also rely on aerobic glycolysis and compete for glucose and produce lactate. We here review the glucose metabolism in the homeostasis of the outer retina and in macrophages and hypothesize what happens when the metabolism of photoreceptors and the RPE is disturbed by chronic inflammation.

## 1. Introduction

All cellular functions rely intimately on energy metabolism. In neurons in particular, this energy demand, in the form of energy-storage adenosine triphosphate (ATP), is met by glucose metabolism. Glycolysis, an oxygen-independent pathway, results in the transformation of one 6-carbon glucose molecule in two 3-carbon pyruvate molecules, two molecules of reduced nicotinamide adenine dinucleotide (NADH), and two ATP molecules, additionally to protons and water molecules [1]. Glycolysis thereby produces energy in the second part of its 10-step pathway. Its energy-consuming first five preparatory chemical reactions also produce intermediates such as dihydroxyacetone phosphate (DHAP), which is the source of glycerol that forms the hydrophilic head of phospholipids, the main constituent of cell membranes. Glucose-6-phosphate (G6P), the first intermediate of glycolysis, can also enter the pentose phosphate pathway (PPP) that generates nicotinamide adenine dinucleotide phosphate (NADPH) and pentoses (5-carbon sugars). Pyruvate, the end product of glycolysis, is converted to acetyl coenzyme A (acetyl-CoA) which, among others, participates in lipid synthesis. Most importantly, in the mitochondria, acetyl CoA delivers the 2-carbon acetyl to the eight-step tricarboxylic acid cycle (TCA) that produces the gradient of protons across the inner membrane of mitochondria which drives the production of ATP in a process called oxidative phosphorylation (OXPHO). The oxidation of one glucose molecule through glycolysis, TCA, and OXPHO theoretically generates 38 ATP molecules, which far exceeds the yield of glycolysis only. Under aerobic conditions and in the presence of mitochondria, most eukaryotic cells therefore produce energy through glycolysis, TCA, and OXPHO, while they are constrained to rely on glycolysis only in anaerobic conditions. In anaerobic glycolysis, to keep the glycolysis reactions going, pyruvate, the end product of the pathway, is reduced to lactate, which is excreted from the cells [2].

When carbon demand is high to synthesize proteins, carbohydrates, and lipid membranes in growing or proliferating cells (such as cancer cells, but also photoreceptors that constantly renew their outer segments) glycolysis exceeds the demand of pyruvate for the TCA in order to produce intermediary metabolites and extra energy in a process called aerobic glycolysis or the Warburg effect [3]. Additionally, it has become increasingly clear that immune cells and, in particular, inflammatory macrophages undergo profound metabolic reprogramming in which the TCA ceases to produce energy but its intermediary products are used for immunological functions. The cells therefore rely on aerobic glycolysis and secrete lactate [4,5].

Physiologically, glucose metabolism by cones, rods, and the retinal pigment epithelium (RPE) are linked in an intricate play of glucose uptake, aerobic glycolysis, and lactate metabolism, essential for the homeostatic production of photoreceptor outer segments and visual function. In age-related macular degeneration (AMD) the photoreceptor cell layer, which is physiologically devoid of even resident macrophages, becomes chronically infiltrated by inflammatory macrophages [6,7] with their special demands in glucose and their important lactate production. To date, little is known about the metabolic disturbances that likely reign in the retina of AMD patients and whether and how they influence the pathogenesis. In this review, we summarize the current knowledge of the homeostatic metabolism of the outer retina, the metabolism of inflammatory macrophages, and what might be the consequence when chronic inflammatory macrophages disturb the metabolic exchanges of photoreceptors and the RPE in diseases such as AMD.

## 2. Photoreceptor Metabolism and Rod-derived Cone Viability Factor

**Figure 1:**
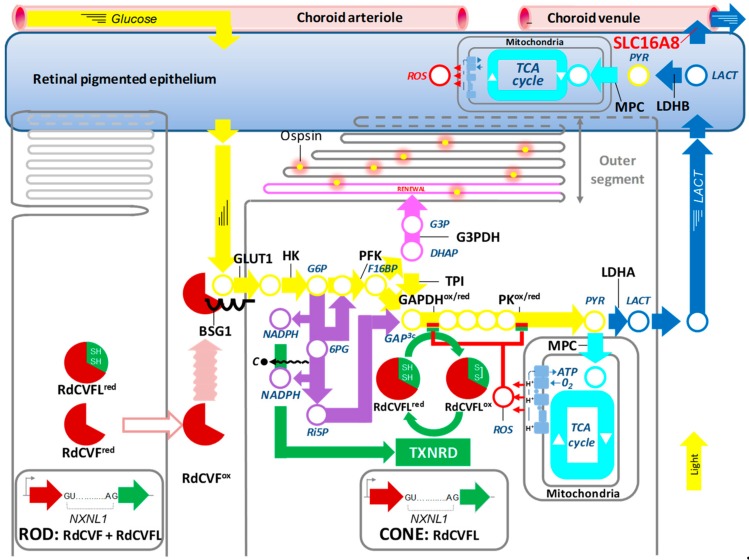
Metabolic and redox signaling regulated by the nucleoredoxin-like 1 gene products. 6PG: 6-phosphogluconate, BSG1: basigin-1, DHAP: dihydroxyacetone phosphate, F16BP: fructose-1,6-biphosphate, GLUT1: glucose transporter SLC2A1, G3P: Glycerol-3-phsopahe, G6P: glucose-6-phospate, G3PDH: glycerol-3-phosphate dehydrogenase, GAPDH: glyreraldeheyde-3-phosphate dehydrogenase, HK: hexokinase, LACT: lactate, LDHA: lactate dehydrogenase A, LDHAB: lactate dehydrogenase B, MPC: mitochondrial pyruvate carrier, NADPH: nicotinamide adenine dinucleotide phosphate, NXNL1: nucleoredoxin-like 1, PEP: phosphoenol pyruvate, PK: pyruvate kinase, PYR: pyruvate, PFK: phosphofructokinase, RdCVF: rod-derived cone viability factor (trophic factor), RdCVFL (thioredoxin enzyme), Ri5P: ribulose-5-phsophate, ROS: reactive oxygen species, SLC16A8: lactate transporter MCT3, TCA: tricarboxylic acid cycle, TPI: triosephosphate isomerase, TXNRD: thioredoxin reductase, ^red^: reduced, ^ox^: oxidized.

RdCVF is an inactive thioredoxin secreted by rod photoreceptors that protects cones from degeneration. It is encoded by the nucleoredoxin-like 1 (*NXNL1*) gene. It does not function as a redox protein as it is truncated in its thioredoxin motif and is consequently not enzymatically active, even with the two catalytic cysteines normally present (CxxC). The reason is that to work as an enzyme, these cysteines must be regenerated (recycling oxidized > reduced) after the thiol-oxidoreductase reaction by thioredoxin reductase. Thioredoxins are secreted by an unconventional pathway that does not rely on the N-terminal leader sequence and are not sorted through the endoplasmic reticulum [8]. A cell surface receptor for RdCVF was identified by far-Western blotting [9]. The single-pass transmembrane protein basigin (BSG) is expressed in two different isoforms. The protein BSG2 possesses two extracellular immunoglobulin (Ig) domains and is expressed widely in many organs where it is involved in trafficking lactate transporters to the membrane. The protein BSG1, produced by alternative splicing, possesses a third Ig domain and its expression is restricted to photoreceptors [10]. RdCVF interacts specifically with a complex formed between BSG1 and the glucose transporter GLUT1 (SLC2A1) at the surface on the cones. RdCVF stimulates that transport activity of GLUT1, most likely by triggering its tetramerization, which relies on a reversible redox-dependent interconversion. Accessible cysteine residues in GLUT1 (SLC2A1) would be oxidized by the extracellular and oxidized form of RdCVF (RdCVF^ox^) that would act as a prooxidant. Oxidized thioredoxins are pro-oxidant, as is the protein disulfide isomerase, another thioredoxin enzyme that catalyzes the formation of disulfide bridges (oxidation of two cysteines) in proteins transiting through the endoplasmic reticulum [11,12,13]. Increased glucose entry into the cones is metabolized by aerobic glycolysis [3]. The bifurcation from oxidative phosphorylation takes place at the level of pyruvate (Figure 1●). For OXPHO, the pyruvate (PYR) can be transported to the mitochondria by the mitochondrial pyruvate carrier (MPC) where it is metabolized by the tricarboxylic acid (TCA) cycle, which is linked to the mitochondrial respiratory chain, to produce ATP in aerobic conditions (Figure 1●>●). Alternatively, the pyruvate can be converted to lactate (LACT) by the enzyme lactate dehydrogenase (LDHA). Lactate is then transported out of the cell by the lactate transporter MCT1 (SLC16A1) (Figure 1●). The phenomenon of aerobic glycolysis was discovered by Otto Warburg as specific to cancer cells that prioritize the production of lactate over the production of ATP by OXPHO even in the presence of oxygen^2^. He also observed that the retina, like cancer cells, produces large amounts of lactate by metabolizing glucose through aerobic glycolysis. RdCVF stimulates aerobic glycolysis to provide the intermediates required for outer segment renewal. This is a monumental task since 10% of the outer segment is renewed every day and is comparable to the proliferation of cancer cells. For reasons that are only partially known, aerobic glycolysis favors diversification at mid-course of the glycolytic reaction of carbons from glucose at the level of dihydroxyacetone phosphate (DHAP). This 3-carbon metabolite is produced by aldolase (ALDO) by breaking a 6-carbon molecule, fructose-1,6-biphosphate (F16BP) (Figure 1●). The second 3-carbon metabolite produced by ALDO is glyceraldyde-3-phosphate (GAP). Triose phosphate isomerase (TPI) very efficiently converts DHAP into GAP which fuels the glycolysis downstream, but a certain proportion of DHAP is converted into glycerol-3-phosphate (G3P) by glycerol-3-phosphate dehydrogenase (G3PDH) (Figure 1●). G3P is the precursor of the glycerol backbone of phospholipids. The insulin/mammalian target of rapamycin signaling is required at a distinct level in the stimulation of aerobic glycolysis in the cones [14,15]. Hypoxia-inducible factor 1-alpha (HIF1A) increases the expression of GLUT1 (SLC2A1) in the cones [14]. The metabolic stimulation by RdCVF relies on a protein signal whose expression is regulated by insulin. Recently, it was demonstrated experimentally that in rods, aerobic glycolysis is necessary for outer segment rod outer segment renewal, a phenomenon parallel to that of cones [16]. Lactate produced by aerobic glycolysis is transported out of the cone photoreceptors by MCT1 (SLC16A1) [17] and cleared from the subretinal space by the retinal pigmented epithelium (RPE) via MCT1 located on its apical side (toward the photoreceptors). The lactate produced by the photoreceptors is converted by the RPE to pyruvate by the LDHB and fuels the oxidative phosphorylation and ATP production in the RPE [18]. As the RPE meets its energy demands by the lactate produced by the photoreceptors, its consumption of glucose is low, which enables the RPE to transport the glucose trans-epithelially from the choroid to the photoreceptors. Excessive lactate is evacuated from the RPE through the lactate transporter MCT3 (SLC16A8) located on its basal side (toward the choroid) [19] (Figure 1●). A deficit in lactate clearance, as observed in the *Slc16a8^−/−^* mouse, will lead to a build-up of lactate in the RPE and the inter-photoreceptor matrix (the space between photoreceptors) and ultimately counteract the efflux of lactate from the cones, which will impair cone aerobic glycolysis and cone outer segment renewal cone function in the central retina [20]. Taken together, aerobic glycolysis in photoreceptors serves to produce G3P to renew their outer segments and to make lactate to feed the RPE in this metabolic ecosystem.

The role of the products of the *Nxnl1* gene was also explored in cones. The retina of a mouse with a specific deletion of the *Nxnl1* in cones is more susceptive to oxidative damage [21]. Not surprisingly, *Nxnl1* is also expressed by cones (3% of all photoreceptors in the mouse). Contrarily to the rods, there is no intron retention in the cones and, consequently, they express only the thioredoxin RdCVFL. Reactive oxygen species (ROS) are produced in physiological conditions by leakage from the mitochondrial respiratory chain (Figure 1●>●). These reactive molecules can interfere with the flux of glucose because two enzymes, glyceraldehyde-3-dehydrogenase (GAPDH) and pyruvate kinase (PK), contain cysteine residues in the catalytic domain or in a regulatory region, respectively. These residues are prone to oxidation by ROS, and, consequently, GAP is accumulating (Figure 1●/●). The glycolytic enzymes are highly allosterically regulated; the accumulation of the product of one reaction inhibits the enzyme that is responsible for its synthesis. Therefore, the accumulation of GAP triggers the accumulation of glucose-6-phosphate (G6P) (Figure 1●<). The flux of carbon from glucose is diverted to the pentose phosphate pathway (PPP) producing ribulose-5-phosphate (Ri5P) by the loss of one carbon molecule (**C**●) and the reduction of two molecules of nicotinamide adenine dinucleotide phosphate (NADP^+^) into NADPH, which provides reducing power (Figure 1●>●). Both 6-phosphogluconate (6PG) and Ri5P can reenter the glycolytic pathway if the inhibition by cysteine oxidation of downstream glycolytic enzymes is alleviated. Otherwise, the metabolites re-enter a second round of the PPP while losing one carbon originating from 6-carbon glucose at every cycle, so in case of prolonged oxidative stress, all the carbon atoms of glucose are oxidized into CO_2_ to provide more reducing power through NADPH.

The thioredoxin enzymes, including RdCVFL, must be reduced by thioredoxin reductase (TXNRD) since the catalytic cysteines become oxidized when the enzyme reduces its substrate (here, GAPDH and PK) [22] (Figure 1●). TXNRD requires its co-factor NADPH, produced by the PPP in its reduced form, to function. Consequently, the reducing power of RdCVFL in cones is proportional to the uptake of glucose and the rederivation of the glycolytic flux to the PPP (Figure 1●>●>●). It is not yet proven that RdCVFL reduces GAPDH and PK, but RdCVFL may interact with PK [23].

When rods have been lost by a direct effect of a mutation causing retinitis pigmentosa, oxygen is still reaching the outer retina from the RPE, since blood flow from the underlying choroid blood beneath the RPE is not regulated by O_2_ consumption in the outer retina, the layer of the retina containing photoreceptor cells [24,25]. The loss of 95% of the photoreceptors (rods) that were consuming oxygen creates hyperoxic conditions for the remaining cones (5%). The cones are highly sensitive to oxidative damage [26]. The combined activities of the *NXNL1* gene products on cones explain rather satisfactorily the clinical observations. The absence of RdCVF caused by rod degeneration in retinitis pigmentosa reduces the uptake of glucose by cones, resulting in the reduction of the size of their outer segments, leading progressively to central blindness. The cones are not dying but persist as light-insensitive cones in patients for years [27]. These cones are exposed to hyperoxia and they require the action of the reducing power of RdCVFL relying on the supply of NADPH through the PPP to survive. Because this supply is reduced by lack of expression of RdCVF by rods, the cones ultimately die. In this scenario, there is a time window for cones to regenerate their outer segment if the expression of RdCVF could be restored. Regeneration of cone outer segments restoring cone visual function has been observed in a pig model of dominant retinitis pigmentosa after local administration of glucose [28]. Intriguingly, spontaneous regrowth of cone outer segments has also been reported in a patient with acute idiopathic blind spot enlargement, demonstrating that the damaged cones can have the capability to recover in human disease [29].

Although the *NXNL1* gene does not contain risk alleles for age-related macular degeneration (AMD), the fact that the *NXNL1* gene encodes for two protein products that protect the cones at the center of the macula whose function is ultimately defective in AMD could suggest that the *NXNL1* gene is involved in AMD pathophysiology. In addition, the role of one of the products of the gene, the thioredoxin RdCVFL, in repairing oxidative damage to the cones is a potential connection with the maintenance of cone function during the aging process.

## 3. Age-related Macular Degeneration

AMD is a complex disorder that affects primarily the center of the retina that contains a region enriched in cone photoreceptors, the fovea. Daylight vision, color perception, and visual acuity are mediated by the function of cones. Early AMD is characterized by sizeable deposits of lipoproteinaceous debris called soft drusen that can be complicated by choroidal neovascularization (wet AMD, late form) or by an extending lesion of the RPE and photoreceptors (geographic atrophy, GA, late form) [30]. It is a late-onset disease resulting from the interplay of age and multiple genetic susceptibility genes and environmental factors, but its pathogenesis remains, by and large, obscure. Late AMD is the principal cause of irreversible visual impairment in the elderly. The estimated prevalence of advanced AMD is of 0.2% at ages 55 to 64 years but increases to 13% in those older than 85 years. Due to the aging population we must therefore expect a sharp increase in in the number of patients with AMD. To date, it is not clear how aging and AMD risk factors trigger the pathogenesis. Choroidal involution, non-resolving low-grade inflammation, and RPE dysfunction and resulting defective photoreceptor outer segment renewal are the prominent proposed hypotheses for its pathogenesis [30].

The earliest accounts of AMD in 1855 and 1868 were linked to the observation of drusen associated with visual loss [31]. Small deposits, called hard drusen (<63 mm in diameter) are associated with normal aging. However, large, soft drusen (>125 mm in diameter) contain lipids, cellular debris, and at least 129 distinct proteins and are enriched in carboxyethylpyrrole protein adducts, biomarkers of oxidative stress which are associated with AMD [32,33]. It is not clear whether the constituents of drusen are solely derived from the RPE, choroidal vasculature, or both. Eyes with large-sized soft drusen can progress and develop late AMD (~15% in the Beaver Dam study over 10 years; ~30% in the Blue Mountain study over 6 years), regress (~25%, Beaver Dam study), or stay stable for years [34,35]. Soft drusen therefore represent an important ocular risk factor to develop late AMD. Additionally, reticular pseudodrusen are strongly associated with AMD [36,37]. They are ~30–150 μm pale fundus lesions that are believed to be caused by subretinal drusenoid deposits located between the RPE and photoreceptors [38,39,40].

In the atrophic late form of AMD (GA), an extending atrophic zone forms that is characterized by the loss of the RPE and degeneration of the photoreceptors [41]. In the atrophic zone, despite the absence of RPE, residual cones (and, to a lesser extent, rods) survive, but they lack their inner and outer cone outer segments necessary for light perception [42,43]. In a perilesional transitional zone, directly peripheral to the area of RPE loss of the atrophic zone, the number of rods drops dramatically compared to regions more distant from the lesion, despite the presence of the RPE [42,43,44]. These anatomical changes translate clinically to decreased perilesional retinal sensitivity [45]. The number of cones changes little in the transitional zone, but they lack their outer segments [42,43,44]. One could assume that cone outer segment loss in the transitional zone of AMD patients is due to a primary RPE dysfunction; however, cone outer segment loss is also observed in patients with retinitis pigmentosa after rod degeneration and unremarkable RPE change [46]. Clinically, GA lesions provoke central scotomas, which severely affect visual acuity when the lesion involves the fovea itself. Patients with late AMD also suffer from more general impaired dark adaptation and increased recovery time after bleach, suggesting a slowing of the vitamin A cycle [47,48]. Interestingly, a significant increase in recovery time after bleach is already observed with reticular pseudodrusen in early AMD [47,48] and is associated with an increased incidence of AMD [49], and this is quite some time before visible RPE or photoreceptor lesions occur. There is currently no therapy that has been approved for atrophic AMD. 

Choroidal neovascularization (CNV) characterizes the second subtype of late AMD, known as wet AMD [50]. CNV occurs for the most part by the growth of new blood vessels through Bruch’s membrane, but in about 10%–15% of exudative AMD, subretinal neovascularization arises intra-retinally from the retinal vasculature (retinal angiomatous proliferations) [51,52]. Interestingly, eyes with CNV also present a loss of choriocapillaris in the surrounding areas [53]. The leaky neo-vessels are commonly associated with retinal edema, subretinal exudation, and blood and lipid deposits leading to rapid loss of vision. If left untreated, a neovascular fibrous membrane and disciform subretinal scar will form [41]. The development of the anti-angiogenic therapy anti-vascular endothelial growth factor (VEGF), capable of controlling the permeability and growth of neovessels, has dramatically improved the treatment for neovascular AMD. It does not, however, halt vessel-independent degenerative processes and the decline in visual functions that occurs in the long term in 30% of patients [54].

## 4. Chronic Low-Grade Inflammation in AMD

Inflammation is a crucial process for survival even after minor skin or mucosa injuries that frequently occur throughout our life. If the ensuing inflammation does not eradicate or circumscribe the invasion of microbes that invariably accompany tissue injury and define infection, their proliferation will quickly become life threatening. The inflammatory process is typically triggered by the activation of resident macrophages and mast cells, followed quickly by the recruitment of neutrophils and blood monocytes which differentiate into inflammatory macrophages. Both cell types express high levels of toll-like receptors (TLRs) that bind pathogen-associated and damage-associated molecules, which activates the transcription of a number of inflammatory mediators. The activated macrophages kill, phagocytose, and eliminate the invading commensal flora and pathogens and alter the tissue to facilitate the immune response (vasodilation, increased permeability, increased sensitivity) by secreting inflammatory cytokines, such as interleukin-1β (IL1B), tumor necrosis factor-α (TNF), IL6, and C–C motif chemokine 2 (CCL2) [55]. Once disinfected, neutrophils of the site of injury/infection undergo death within hours and are cleared together with tissue debris by macrophages. Phagocytosis of dead neutrophils and other stimuli polarize the macrophages to facilitate tissue repair, scar formation, and inflammation resolution [56,57]. Finally, the inflammatory macrophages die by apoptosis and the tissue is left with the tissue-specific resident macrophages, as before the injury [58].

In order to re-establish tissue homeostasis, the inflammatory reaction needs to rapidly and efficiently resolve. If the inflammatory response is not quickly controlled, it can become pathogenic. Non-resolving low-grade inflammation contributes significantly to the pathogenesis of many chronic, age-related diseases. It is not a primary cause of these diseases, but it contributes significantly to their pathogenesis as it can also cause considerable collateral damage fueling further inflammation [59]. In the affected tissues, it is often associated with the persistent recruitment and presence of monocyte-derived inflammatory macrophages [59]. 

AMD is a typical example of chronic inflammation. It is associated with non-resolving subretinal accumulation of macrophages around large drusen in intermediate AMD, where they might fulfill a homeostatic role, controlling debris accumulation and drusen growth [60,61]. Macrophage accumulation is also observed in both advanced forms [30,43,60,61,62], where they are a chronic source of inflammatory cytokines [63]. The infiltrate in atrophic late AMD invariably contains inflammatory macrophages [61]. Moreover, the intraocular concentrations of CCL2 are increased in patients with atrophic (but also in neovascular) AMD [61,64,65,66]. In animal models, blocking inflammatory macrophage recruitment, or inhibiting their TLR signaling, dramatically reduces CNV [67,68] and photoreceptor degeneration [61,69,70,71,72]. This is likely due to the fact that inflammatory macrophages are an abundant source of pathogenic inflammatory cytokines such as IL1B, involved in rod and cone degeneration [43,73] and CNV [74]; TNF, which inhibits RPE visual cycle genes [75]; and IL6, which diminishes RPE immunosuppressivity [60] and participates in CNV [76]. Accordingly, the accumulation of macrophages in the transitional zone (surrounding the atrophic lesions of GA patients) is closely associated with rod death and cone outer segment loss [43].

## 5. Inflammation-Induced Metabolic Changes in AMD

**Figure 2:**
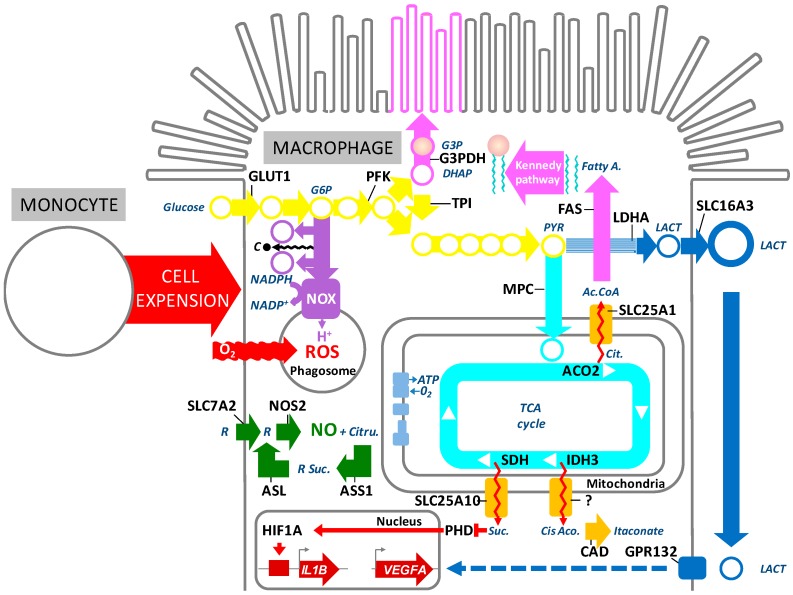
Metabolic reprogramming of inflammatory macrophages. Ac.CoA: acetyl-coenzyme A ACO2: aconitase mitochondrial, ASL: argininosuccinate lyase, ASS1: argininosuccinate synthase 1, CAD: *cis*-aconitate decarboxylase, *Cis*-Aco.: *cis*-aconitate, Cit.: citrate, Citru.: citrulline, DHAP: dihydroxyacetone phosphate, Fatty A.: fatty acids, FAS; Fatty acid synthase, G3P: Glycerol-3-phsopahe, G3PDH: glycerol-3-phosphate dehydrogenase, G6P: glucose-6-phospate, GLUT1: glucose transporter SLC2A1, HIF1A: hypoxia-inducible factor 1, IDH3: isocitrate dehydrogenase 3, IL1B: interleukine-1β, IRG1: immune-responsive gene 1, LACT: lactate, LDHA: lactate dehydrogenase A, MPC: mitochondrial pyruvate carrier, NADPH: nicotinamide adenine dinucleotide phosphate, NO: nitric oxide, NOS2: inducible nitric oxide synthase, NOX: NADPH oxidase, PFK: phosphofructokinase, PHD: prolyl-hydroxylase, PK: pyruvate kinase PYR: pyruvate, R: arginine, ROS: reactive oxygen species, SDH: succinate dehydrogenase, Suc.: succinate, SLC7A2: arginine transporter, SLC16A3, lactate transporter MCT4, SLC25A10: mitochondrial dicarboxylate carrier, TCA: tricarboxylic acid cycle, TPI: triosephosphate isomerase, VEGF: vascular endothelial growth factor.

Recruitment and activation of macrophages in the transition zone in AMD would be expected to profoundly change the metabolic environment. Indeed, monocyte-derived inflammatory macrophages undergo a fascinating metabolic reprogramming (Figure 2). Upon activation, the TCA cycle of macrophages is interrupted at several levels and the accumulating TCA intermediates are used for the production of anti-microbial mediators and to activate pathways necessary for the function of inflammatory macrophages [77]. In turn, glucose uptake and aerobic glycolysis increase to meet the energy demand and generate metabolic intermediates needed for cell growth (Figure 2●/●). The pentose phosphate pathway (PPP) produces NADPH which is utilized by NADPH oxidase (NOX2) to reduce molecular oxygen to produce superoxide (O−2O2−) that is then further dismutated into stable and diffusible hydrogen peroxide pro-oxidan for bactericidal functions [4,5,78,79] (Figure 2●/●). MCT4 (SLC16A3) facilitates the efflux of lactate from macrophages to maintain a high rate of glycolytic flux (Figure 2●).

This metabolic switch in macrophages is likely regulated on several levels. TLR activation participates in the induction of inducible nitric oxide synthase (NOS2), which is a key enzyme of inflammatory macrophages. NOS2 metabolizes arginine to nitric oxide (NO) and citrulline, which might be reused for NO synthesis via the citrulline–NO cycle [80] (Figure 2●). NO is a highly reactive molecule that diffuses freely through membranes and can be metabolized to further downstream reactive nitrogen species that can alter enzyme function by nitrosylation. Although NO production from human monocyte-derived macrophages in vitro is controversial [81], its detection in circulating monocytes (and increased production in arthritis) [82] and NOS2 expression in macrophages in AMD [83] suggest that it is possibly involved in the metabolic shift of inflammatory macrophages in AMD [82,83]. Nitric oxide, released from NOS2, can impede the function of a number of enzymes of the TCA cycle by S-nitrosylation, including the isocitrate dehydrogenase (IDH3, which catalyzes the decarbolxylation of isocitrate to alpha-ketogluterate), succinate dehydrogenase (SDH, which oxidizes succinate), and aconitase (ACO2, which catalyzes the isomerization from citrate to isocitrate via *cis*-aconitate) [84]. The inhibition of enzymes in the TCA cycle results in an accumulation of citrate, *cis*-aconitate, and succinate in the mitochondria.

The mitochondrial citrate carrier (SLC25A10) exports citrate, an intermediary metabolite of the TCA cycle, outside the mitochondrial matrix in exchange for malate. Citrate is metabolized by ATP-citrate lyase (ACLY) to acetyl-CoA and oxaloacetate in the cytosol. Acetyl-CoA carboxylase (ACACA) metabolizes the acetyl-CoA into malonyl-CoA, which is used to produce fatty acids by fatty acid synthase (FAS). Fatty acids are combined with glycerol (produced from G3P that is derived from the glycolysis-intermediate DHAP) to form phospholipids by the Kennedy pathway necessary for membrane synthesis. Indeed, when the small monocyte precursor differentiates into a large, secretory, inflammatory macrophage, it needs to increase its production of membranes to form the long pseudopods on its surface and intracellular phagosomes and lysosomes [78,85,86,87] (Figure 2●>●●). 

*Cis*-aconitate is transported from the mitochondria to the cytoplasm where it is metabolized to itaconate by the *cis*-aconitate decarboxylase (CAD) encoded by the immune-responsive gene 1 (*IRG1*) which is highly induced by TLR activation in inflammatory macrophages [5,88] (Figure 2●>●). While itaconate is best known for its capacity to inhibit the prokaryotic isocitrate lyase necessary for bacterial feeding on acetate and fatty acid [89], it also acts as a competitive inhibitor of succinate dehydrogenase (SDH), which will further increase succinate accumulation [5,90,91].

Succinate, in turn, is transported to the cytosol via the mitochondrial dicarboxylate carrier (SCL2A10), where it leads to the accumulation of hypoxia-inducible factor 1 (HIF1A) by inhibiting prolyl-hydroxylases (PHDs) and thus preventing HIF1A hydroxylation and degradation [92] (Figure 2●>●●). Increased stability of HIF1A induces a state of pseudohypoxia [92], pushing aerobic glycolysis for energy production and, consequently, lactate production. Interestingly, succinate-induced HIF1A stabilization is also sufficient to induce IL1B mRNA expression and its maturation through the inflammasome [93,94]. Additionally, succinate and lactate can induce cytokine and VEGF secretion in macrophages through their respective receptors, GPR91 and GPR132 [95,96,97] (Figure 2●●). Interestingly, the lactate transporter MCT4 (SLC16A3) is required for metabolic reprogramming and inflammatory response in macrophages [98,99].

Additionally, TLR activation induces G6PDH, the rate-limiting enzyme of the PPP, which produces ROS that are indispensable for the intra-lysosomal killing of pathogens in activated macrophages [5,100] (Figure 2●/●, see above).

The metabolic switch of inflammatory macrophages to aerobic glycolysis makes them very reliant on the surrounding glucose concentrations for survival and function. This demand is met in part by the IL1B- and TNF-mediated induction of insulin resistance in adjacent cells, thereby decreasing their competing glucose consumption [101,102]. This mechanism is increasingly recognized to play an important role in insulin resistance in the adipose tissue of type 2 diabetes [101], but has also been suggested to play in important role in neuronal death in uveitis, a blinding auto-immune disease of the retina and choroid [103].

The metabolism of inflammatory macrophages reveals two elements to be noted which differentiate them from the situation of the cones described. First, the use of NADPH as a molecule involved in the production of bactericidal ROS, additionally to the repair of oxidation damage to cysteines and methionines. Second, the TCA cycle is disrupted in inflammatory macrophages and does not serve for energy production, which is not the case for cones whose function as neurons also depends on ATP production by the mitochondrial respiratory chain. These differences might also reflect the vast discrepancies of life span in both cells. Inflammatory macrophages live on aerobic glycolysis, interrupt their OXPHO, and are characterized by TCA intermediate accumulation. They burn their candle at both ends, so to speak, have a very short half-life, and are eliminated by apoptosis during the resolution of inflammation. Cones metabolize glucose through aerobic glycolysis for outer segment renewal and OXPHO for ATP production but do not synthesize fatty acids de novo and live for decades [104]. Cancer cells stimulate the metabolism of glucose through aerobic glycolysis and OXPHO, which is closer to the situation of the cones. Similar to cones, the cancer cells have a longer life span and are not quickly eliminated by apoptosis [105].

## 6. Genetic Susceptibility for Subretinal Metabolic Changes

Age and a positive family history of AMD are the two strongest risk factors for AMD. It has been shown that an individual with a sibling or a parent with AMD is 12–27 times more susceptible than someone without to develop AMD. Genome-wide association studies (GWAS) have led to the identification of several AMD susceptibility alleles in a total of 34 genes [106,107].

The apolipoprotein E (APOE) alleles, along with a common variant of complement factor H (CFH) and a risk haplotype on chromosome 10q26, account for most of the genetic risk to late AMD^30^. *APOE2*-allele carriers are at increased risk, while the ancestral *APOE4*-allele protects against AMD compared to the most common *APOE3*-allele [30,108]. High levels of APOE expression are observed in subretinal mononuclear phagocytes of AMD patients [60], CX3C chemokine receptor 1 (CX3CR1)-deficient mice^60^, and humanized transgenic mice expressing the human AMD risk *APOE2*-allele (TRE2 mice) [60,61,109]. Excessive expression of APOE activates the cluster differentiation (CD)14/TLR2/TLR4-dependent innate immunity receptor complex (IIRC) on the surface of macrophages [60,109], likely by modifying the cholesterol content of the lipid raft in which the receptor complex is localized [30]. Its activation in subretinal macrophages induces CCL2 [109], which increases monocyte recruitment [61], but also IL6, which reduces FAS ligand (TNFSF6) expression by the RPE and macrophage elimination [60]. Consequently, APOE induces chronic, age-related and pathogenic subretinal macrophage accumulation in CX3CR1-deficient and TRE2 mice [60,61,109]. In the context of APOE-dependent subretinal inflammation of *Cx3cr1^GFP/GFP^* mice, the *APOE4*-allele decreases APOE and CCL2 expression levels and protects *Cx3cr1^GFP/GFP^* mice against harmful subretinal mononuclear phagocyte accumulation observed in *Cx3cr1^GFP/GFP^* [109]. These studies demonstrate how APOE2 provokes and APOE4 inhibits the accumulation of pathogenic inflammatory macrophages in AMD.

Another important genetic risk factor for AMD is a variant of complement factor H (CFH) formed by the substitution of tyrosine 402 to histidine (Y402H) in the seventh short consensus repeat (SCR7) domain of CFH [30]. CFH is expressed at high levels in the microglial cells and monocyte-derived macrophages that infiltrate the subretinal space in AMD. CFH binding to CD11b/CD18 interferes with physiologically occurring thrombospondin 1 (TSP1)/CD47-dependent elimination of macrophages from the subretinal space [110]. Compared to the common CFH^402Y^, the AMD-associated CFH^402H^ is particularly potent at inhibiting the elimination of subretinal microglial cells [110] that produce CCL2 and are responsible for the recruitment of pathogenic inflammatory macrophages in AMD [61].

Taken together, these studies show that both AMD risk variants APOE2 and CFH^402H^ induce the accumulation of subretinal macrophages. In that way, they significantly increase the population of glucose-consuming macrophages producing lactate by aerobic glycolysis. Consequently, the increased subretinal lactate concentration could induce insulin resistance, interfere with the metabolism of photoreceptors, decrease the extracellular pH, and impair visual transduction.

The recently identified risk allele (rs8135665T) within the SLC16A8 gene could also profoundly alter the lactate concentration and pH of the subretinal space [107]. SLC16A8 codes for a transporter that mediates the rapid transport of lactate across the plasma membrane, MCT1 [20]. In the eye, its expression is restricted to the RPE cells, suggesting that its major physiological function in the eye is the transport of lactate produced through aerobic glycolysis by photoreceptors. Lactate is physiologically transported outside cone photoreceptors by MCT1 (SLC16A1) [17], then transported through the RPE by two distinct transporters: MCT1 (SLC16A1) on the apical side (toward photoreceptors) and MCT3 (SLC16A8) on the basal side (toward the choroid) (Figure 1●). The transepithelial transport of lactate is dependent on the “lactate faucets” on both sides of the cell: Since both transporters on each side of the RPE are facilitate transporters, the direction of the flux depends on the difference in lactate concentration between the interphotoreceptor matrix and the cytoplasm of the RPE cell for MCT1 (SLC16A1), and the difference between the lactate concentration in the cytoplasm of the RPE cell and that of the choriocapillaris for MCT3 [111]. A deficit in lactate clearance at the basal side of the RPE will trigger a decrease in transport on the apical side which will result in an increase of lactate concentration in the inter-photoreceptor matrix (the space between photoreceptors); this will reverse the polarity of lactate transport in the cones, counteracting their RdCVF-stimulated physiological aerobic glycolysis. This mechanism likely explains the impaired photoreceptor function and cone damage observed in the *Slc16a8^−/−^* mouse [20] and might be responsible for cone outer segment loss observed in AMD [112]. In addition, the excess lactate metabolized by the TCA cycle in the RPE and leakage from the mitochondrial respiratory chain likely results in the accumulation of ROS and damage in RPE cells [113].

We believe that the lack of efficient lactate transport by the SLC16A8 could be an additional key element in AMD pathogenesis. On one hand, the expression of SLC16A8 protein is decreased in the atrophic area of AMD [114]. On the other hand, an AMD-associated SLC16A8 variant might lead to deficient lactate transport. The candidate causal mutation of SLC16A8 (GT > CT) disrupts a splice site that was also identified but it remains to be firmly genetically linked to AMD [107]. More recently, a genetic analysis revealed that a missense allele in the SLC16A8 protein (R235W), located in the central cytoplasmic loop of the protein, is genetically linked to the AMD risk allele (rs8135665T) [115]. This central intracellular loop of the facilitator superfamily of transport proteins is known to govern efficient membrane insertion during translation [116]. For example, missense mutations in the corresponding region of the glucose transporter SLC2A1 are reported in patients suffering from GLUT1 deficiency syndrome [117]. The implication of R235 in the function of SLC16A8 is presently unknown, but since it is a coding allele it may represent the causative mutation associate with the risk allele rs8135665T and lead to transporter dysfunction, similar to the mutation causing GLUT1 deficiency syndrome.

## 7. Conclusions

We here first reviewed the homeostatic metabolic interactions between retinal cells of the outer retina and highlighted the fundamental role of the menage à trois between rods, cones, and the retinal pigmented epithelium: Glucose is transported through the RPE and taken up in the photoreceptors to produce energy via the TCA cycle and oxidative phosphorylation in the mitochondria. A high rate of glycolysis is needed to produce the DHAP required for glycerol synthesis and the production of phospholipids necessary for the renewal of the outer segments. This aerobic glycolysis additionally leads to efflux from the photoreceptors of lactate which is taken up by the RPE and utilized to support its own energy demand. Excess lactate is transported out of the RPE across the basal lateral membrane by MCT3 (SLC16A8) where it is removed by choroid circulation. Cones rely on the production of RdCVF by rods and insulin signaling for glucose uptake to meet their demand for carbon for outer segment renewal and energy supply. The three cells thereby act as symbiotes: the RPE supplies the glucose from the choroidal circulation to the photoreceptors, the rods help the cones, and both produce lactate to feed the RPE. This intricate play of glucose metabolism between the three cell types is essential for the function and survival of the cells. This interplay might have come about in the evolution of the cone/rod retina, as rods (which appeared after cones in evolution) might have acquired an unknown metabolic advantage over cones concerning the utilization of glucose metabolites for their outer segment renewal. To compensate for this metabolic advantage, an alternative splicing of RdCVF occurred (only) in rods that stimulates glucose uptake via BSG1 in cones, necessary for their function and survival [21,118]. The RPE evolved to efficiently eliminate excessive lactate produced by the photoreceptors, using MCT1 (SLC16A1) and MCT3 (SLC16A8) at its apical and basal sides, which likely differ in their transport capacity. Failure of this sophisticated metabolic ecosystem leads to severe functional and structural consequences in the outer retina.

**Figure 3:**
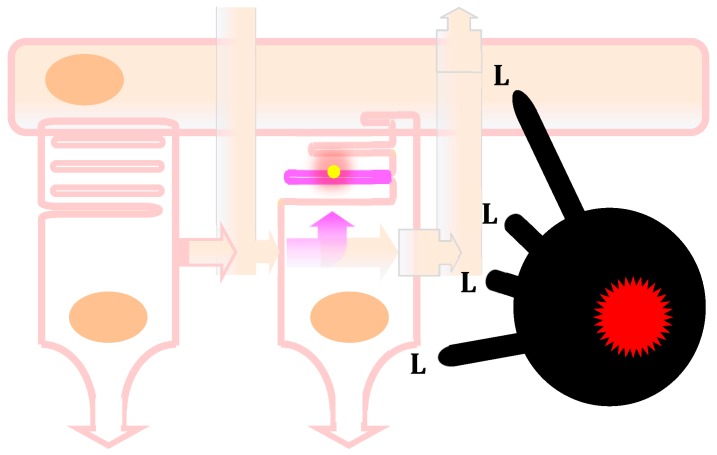
Blood-derived inflammatory macrophage perturbation of the metabolic ecosystem between photoreceptors and the retinal pigmented epithelium. In non-pathological conditions, the rod on the left produces and secretes the truncated thioredoxin rod-derived cone viability factor (RdCVF, pink arrow toward the right). The cone on the right expresses the RdCVF cell-surface receptor basigin-1 (BSG1). RdCVF activates the complex formed between BSG1 and the glucose transporter GLUT1 (SLC2A1) resulting in an acceleration of the entry of glucose coming from the blood circulation through the retinal pigmented epithelium (RPE, on the top). The cone metabolizes glucose through aerobic glycolysis that produces glycerol-3-phosphate as a precursor of the hydrophilic head of the phospholipids (dark pink) for the renewal of the cone outer segment that contains the light-sensing molecule, the opsin (yellow). Aerobic glycolysis also produces lactate that is transported outside the cone by a lactate (lac) transporter. The lactate is partially transported through the RPE toward the blood circulation via two lactate transporters; the one on the basal side (on the top) is encoded by the SLC16A8 gene that carries risk alleles for AMD. A certain proportion of the transported lactate is metabolized by the RPE to pyruvate that fuels the mitochondrial oxidative phosphorylation. In that ecosystem, the glucose issued from the blood circulation is not metabolized by the RPE. In pathological conditions, as in patients carrying SLC16A8 risk alleles for AMD, the accumulation of lactate and its metabolism by the mitochondrial respiratory chain produces an excess of reactive oxygen species by leakage, and since lactate transporters are facilitating transporters, the rise in lactate in the RPE triggers an elevation of lactate in the extracellular space between photoreceptors. For the same mechanistic reason, an excess of lactate outside the cone counteracts the intracellular glycolytic flux and inhibits aerobic glycolysis, resulting in the shortening of the cone outer segment and the impairment of cone vision of the macula. This AMD pathological mechanism, identified through genome-wide association studies, may represent a genetic signature of a role of lactate produced after the metabolism reprogramming of inflammatory macrophages occurring in the disease due to chronic inflammation. This is illustrated by the infiltrated inflammatory macrophage (black, on the far right) which produces lactate and elevates the concentration of lactate in the retina, as does the SLC16A8 risk alleles.

This situation is likely dramatically changed in age-related macular degeneration. Under normal conditions, the photoreceptor cell layer and RPE are devoid of even resident macrophages. In AMD, however, resident and blood-derived inflammatory macrophages chronically infiltrate the photoreceptor cell layer (Figure 3). These innate immune cells have evolved to defend the organism against the existantial thread of infection. Pathogen- and disease-associated molecular patterns activate their toll-like receptors (TLR), which induce the secretion of inflammatory cytokines that induce insulin resistance in the surrounding cells, thereby ensuring the glucose demands of the inflammatory reaction to fend off any potentially deadly pathogen invasion. Additionally, TLR activation in macrophages induces breaks in the TCA that no longer produce energy but utilise TCA intermediates to reprogram the cells to produce bactericidal mediators, cytokines, and cell membranes for phagosomes, lysosomes, and the growth of the monocyte precursor into sizeable macrophages. In turn, inflammatory macrophages produce their energy via aerobic glycolysis that also generates lactate which is transported in their surroundings. The chronic infiltration of the photoreceptor cell layer by inflammatory macrophages is therefore very likely to severely disturb the exchanges of glucose and lactate between the RPE and photoreceptors: the macrophages compete with photoreceptors for glucose; their inflammatory cytokines probably induce insulin resistance in adjacent cells, which could further decrease glucose uptake in photoreceptors; and lactate secretion from the infiltrating macrophages will increase subretinal lactate concentrations, hamper glycolysis in photoreceptors, and produce an increase in ROS in the RPE.

Interestingly, AMD genetic risk factors such as the APOE2 isoform or CFH^402H^ that promote subretinal chronic inflammation should thereby indirectly disturb outer retinal metabolism. More directly, the AMD-associated variant of SCL16A8, which likely leads to insufficient lactate elimination via the choroid, might be particularly overwhelmed in the presence of lactate-secreting macrophages in the subretinal space. The existence of risk alleles for AMD in a lactate transporter expressed exclusively in the retinal pigmented epithelium may be the direct genetic signature for the involvement of metabolic dysfunction as a key mechanism of AMD disease progression.

Our review of the homeostatic metabolic exchanges of photoreceptors and the RPE in addition to the particular metabolic demands of infiltrating macrophages would suggest that the photoreceptor cell layer and subretinal space in AMD is subjected to severe metabolic dysfunctions. Future research is needed to corroborate experimentally whether these changes do take place in the eyes of patients with AMD and to what extent they might be the driving force of the degenerative changes that lead to the loss of vision.

## Abbreviations

6PG	6-phosphogluconate
Acetyl-CoA	Acetyl-coenzyme A
ACO2	Aconitase mitochondrial
ALDO	Aldolase
AMD	Age-related macular degeneration
APO E	Apolipoprotein E
ASL	Argininosuccinate lyase
ASS1	Argininosuccinate synthase 1
ATP	Adenosine triphosphate
BSG	Basigin
BSG1	Basigin splice variant 1
BSG2	Basigin splice variant 2
CAD	*Cis*-aconitate decarboxylase
CCL2	C–C motif chemokine 2
CCR2	CCL2 receptor
CD	Cell determinant
CFH	Complement factor H
CNV	Choroid neovascularization
CX3CR1	CX3C chemokine receptor 1
DHAP	Dihydroxyacetone phosphate
F16BP	Fructose-1,6-biphosphate
G3P	Glycerol-3-phosphate
G3PDH	Glycerol-3-phosphate dehydrogenase
G6P	Glucose-6-phosphate
GA	Geographic atrophy
GAP	Glyceraldyde-3-phosphate
GAPDH	Glyceraldehyde-3 phosphate-dehydrogenase
GLUT1	Glucose transporter 1
GPR91	G-protein coupled receptor 91 (Succinate receptor 1)
GPR132	G-protein coupled receptor 132
HIF1A	Hypoxia-inducible factor 1
IDH3	Isocitrate dehydrogenase 3
Ig	Immunoglobulin
IL1B	Interleukin-1β
IL6	Interleukin-6
IRG1	Immune-responsive gene 1
LACT	Lactate
LDHA	Lactate dehydrogenase A
LDHB	Lactate dehydrogenase B
MPC	Mitochondrial pyruvate carrier
NADH	Nicotinamide adenine dinucleotide (reduced)
NADP^+^	Nicotinamide adenine dinucleotide phosphate (oxidized)
NADPH	Nicotinamide adenine dinucleotide phosphate (reduced)
NOS2	Inducible nitric oxide synthase
NOX	NADPH oxidase
NXNL1	Nucleoredoxin-like 1
OXPHO	Oxidative phosphorylation
PHD	Prolyl-hydroxylase
PK	Pyruvate kinase
PPP	Pentose phosphate pathway
PUFA	Poly-unsaturated fatty acids
PYR	Pyruvate
RdCVF	Rod-derived cone viability factor
RdCVFL	Rod-derived cone viability factor long (thioredoxin)
RdCVF^ox^	Rod-derived cone viability factor with oxidized cysteines
Ri5P	Ribulose-5-phosphate
ROS	Reactive oxygen species
RPE	Retinal pigmented epithelium
SCR7	Short tandem repeat 7
SDH	Succinate dehydrogenase
SLC16A1	Monocarboxylate transporter 1 (MCT1)
SLC16A3	Monocarboxylate transporter 4 (MCT4)
SLC16A8	Monocarboxylate transporter 3 (MCT3)
SLC25A10	Mitochondrial dicarboxylate carrier
SLC2A1	Glucose transporter 1 (GLUT1)
SLC7A2	Arginine transporter
TCA	Tricarboxylic acid
TLR	Toll-like receptor
TNF	Tumor necrosis factor-α
TNFSF6	FAS ligand
TPI	Triose phosphate isomerase
TSP1	Thrombospondin 1
TXNRD	Thioredoxin reductase
VEGF	Vascular endothelial growth factor
